# Miscellaneous Intelligence

**Published:** 1810-11

**Authors:** 


### MISCELLANEOUS INTELLIGENCE.

A case of small-pox occurring twice to the same individual, who had also had chicken-pox, has recently come under the observation of Mr. Hewson. The marks which remained on the face, after the first attack of the disease, were very apparent, whilst the pustules of the second were distinct, and were preceded by the eruptive fever in the usual way. Mr. Hewson inoculated two children with some of the matter; one of them, an infant at the breast, had only "a slight sprinkle of pustules, the other had a large crop of distinct small-pox, which began to turn about the ninth day." If care were taken to record them, such instances would not appear rare. An intelligent observer has remarked, that where small-pox has occurred after vaccination, the disease has uniformly been mild; but in general, when small-pox has affected the same patient twice, the second attack is severe.

The purgative method of curing fever, so strongly recommended by Dr. Hamilton of Edinburgh, is gaining ground in this metropolis. It has proved eminently successful in Scarlatina, as well as in Typhus.—Bark, acids, and wine, are now resorted to by many practitioners, only when the febrile action has ceased, to restore the strength of the patient.

Adam Burt, M. D. Superintendant Surgeon, Muttra, has discovered a blistering fly, a species of Meloe, which possesses blistering powers much superior to those of the Spanish fly in common use. As specimens of it have been sent to Sir Joseph Banks, we may hope shortly to be favoured with a more enlarged account of an insect, which promises to become a valuable addition to our Materia Medica.

From the French Gazette de Santé, we learn, that when the horse-chesnut is ripe, and drops from the tree, if we strip off the brown husk which covers it, and pound the kernel in a mortar, then apply the farina thus obtained to the soiled parts of linen, and afterwards wash it, the spots will disappear as completely as if soap had been used.

About the period when the *Eau Medicinale D'Husson*, after having flowed its course in France, was introduced into this country, the Parisians were entertained with wonderful accounts of an "*Eau de Pradier*," or "*Topique anti-arthritique*." The liquid is directed to be poured poured on a linseed meal poultice, with which the limbs, especially the legs of the gouty patient are to be covered. The effects of this application are, the skin becomes soft and moist; a humid exudation of a white colour bedews the surface, and some of it generally adheres to the poultice. A painful burning sensation is felt in the sole of the foot, and in the heel; this sometimes increases to a violent degree, although redness and inflammation do not occur. When the applications have been often repeated, weakness and emaciation of the legs take place; the soles of the feet become extremely tender; and some persons are affected with general agitation, restlessness, and increased activity in all the functions. Mons. Pradier asserted, that the composition was purely vegetable, and defied any chemical tests to analyse it; but offered to reveal the secret for a considerable sum of money. Many people tried the remedy, and, as usual, some of them were relieved, whilst others suffered sufficiently to excite alarm in the profession. In one journal, Pradier was abused, and his remedy vilified, whilst in another their merits only were attested.

From an analysis of the *Eau anti-arthritique*, in the Gazette de Santé for November 1809, it appears to consist of a spirituous infusion of saffron, lime-water, and *gum-guttæ*, a production of a tree called *Coddam-pulli.*

---

December 5th, 1809, died suddenly, at Paris, in the 120th year of his age, *Pierre Defournelle*, Doctor in Medicine. This extraordinary man was born at Barjac en Vivarais, the 25th of October, 1690. He enjoyed a pension, from having been a free-mason, one hundred years. He was a great traveller, fond of alchymy, and consumed his patrimony in the pursuit of the *hermetic science.*

---

At the Annual Meeting of the College of Physicians of London, October 1st, the following gentlemen were chosen officers for the ensuing year.

President.—Sir Lucas Pepys, Bart.

Censors.—Drs. Bree, Roberts, Ainsley, and Warren.

Treasurer.—Dr. Budd.

Register.—Dr. Harvey.

Commissioners for Regulating Mad-houses.—Sir Lucas Pepys, Bart. Drs. Stone, Pemberton, Lambe, and Price.

Secretary.—Dr. Powell.

At this Meeting, Drs. Morgan and Simmons were elected Fellows; and Drs. Dwyer and Black Licentiates.

Candidate.—Dr. Agar.

---

Theatre of Anatomy, Blenheim Street, Great Marlborough Street.—Mr. Brookes commenced his Autumnal Course of Lectures on Anatomy, Physiology, and Surgery, on Monday, the 1st of October, at two o'clock.

Spacious apartments, thoroughly ventilated, are open all the morning for the purposes of dissecting and injecting, where Mr. Brookes attends.

Mr. Macartney commenced his Course of Lectures on Comparative Anatomy, and the Laws of Organic Existence, in the Theatre at St. Bartholomew's Hospital, on Monday evening, October the 15th, at eight o'clock. The Lectures will be repeated on the Wednesday, Friday, and Monday evenings, at the same hours during the Course.

---

Middlesex Hospital.—Dr. Satterley's Course of Clinical Instruction will begin the first week in November: the attendance on the patients will be continued daily, and Lectures will be given once a week, or oftener, when it may be necessary, at eleven o'clock. Mr. Cartwright, Surgeon to the Hospital, will undertake such occasional Demonstrations of Morbid Anatomy, as may be required for the illustration of the respective cases. The objects of the Course will also be extended to such remarkable peculiarities in the diseases of Children, as may occur in the Foundling Hospital.

Dr. Young will begin, in February, a Course of Lectures on Physiology, and on the most important parts of the Practice of Physic; in particular the Nature and treatment of Febrile Diseases: he will deliver them on Tuesdays and Fridays, at seven o'clock in the evening.

---

Electrical and Electro-Chemical Science.—Mr. George Singer will commence a comprehensive Series of Lectures on these important branches of Science, early in the ensuing Season. A Prospectus of the plan of instruction may be had of Mr. Cutbertsón, 54, Poland Street, or Mr. Singer, 3, Princes Street, Cavendish Square.

---

Mr. Armiger, late Anatomical Demonstrator at the London Hospital, will commence a course of Lectures on Practical Anatomy and Operative Surgery, on January 1st, 1811. Particulars may be known, by inquiring at 23, Pavement, Moorfields.

---

Lately have been brought to London the skin and some of the bones of that remarkable animal the *Camelopardus Giraffa*. The cranium of this animal, presumed to be the only one in Europe, is in possession of Mr. Brooks, the *Anatomist*; and a few days since was shewn at his Lecture. This cranium is much larger than that of the horse, remarkably delicate in some of its proportions, and has several peculiarities. Some years ago *Lady Strathmore* presented the skin of one of these animals to Mr. John Hunter; there was another at the Hague, which, with that now preparing for the Museum of Mr. Bullock in Piccadilly, are, if we mistake not, the only specimens of this extraordinary creature yet brought to Europe.

---

### BOOKS IN THE PRESS.

A Natural History of the Human Teeth, with a Treatise on their Diseases, from Infancy to Age, adapted for General Information.

mation. To which are added, Observations on the Physiognomy of the Teeth and of the Projecting Chin. By Joseph Murphy, Surgeon Dentist, of Leeds.

A Third and Fourth Part of Mr. Abernethy's Surgical Observation on Injuries of the Head, Miscellaneous Subjects, Chronic Abscesses, and tumours.

A Second Volume of the Medico-Chirurgical Transactions.

Facts and Opinions concerning Diabetes, by Dr. Latham.

A System of Surgery, by Mr. James Russell, Surgeon, F. R. S. &c. and a System of Anatomy, by Dr. John Gordon, of Edinburgh.

The Second Fasciculus of Mr. Watt's Anatomio-Chirurgical Views, intended to illustrate the Anatomy of the Male and Female Pelvis, and their Contents; the Muscles of the Perineum, as they appear on Dissection; and the External Organs of Generation in both Sexes.

A Treatise on Mortification, by Mr. Lee, Surgeon, of Shields.

Mr. Ramsden's Cases of Cure of the Derangements of the Testicle, Demonstration of their being sympathetic with the Urethra, and to shew that most of the Diseases of that Gland are perfectly within Remedy. Also Cases of the Hydrocele, in which the radical Cure has been effected without recourse to any of the Operations at present employed for that Purpose.

A Biographical account of the late Dr. Beddoes, by J. C. Stock, M. D. and a Posthumous Work on the Eye, left by Mr. Saunders.

A Treatise on the Subject of Cancer, by Dr. Denman.

The Seventh Edition of the New London Practice of Physic, by E. G. Clarke, M. D. Author of the Medicinæ Praxeos, &c.

A Concise Description of the Human Bones, chiefly from the Works of the late Professor Monro; accompanied with accurate Engravings.

A new Edition of the Elements of Chemistry, by Murray. This Edition is intended particularly to exhibit the splendid Discoveries of Professor Davy.

A new Edition of Cullen's First Lines of the Practice of Physic, by Dr. Peter Reid, a gentleman of known ability and industry. It is intended to insert the nosology at the head of each chapter, and to improve the whole by the addition of recent discoveries in Medical Science.

---

Mr. W. Salisbury has published the following observations on the probable cause of the destruction of a large proportion of the plane trees in this country last year:—

"There are three different species of *Platanus* commonly cultivated in this country; two are natives of the Levant, and the other of the Northern States of America: those of course differ as much in their habits of growth, as the seasons in the climates which produce them. It is well known to all cultivators of exotic plants, that such as are natives of the colder climates are the earliest earliest in vegetating, being most sensibly acted on and forced forward by the mildness of the weather commonly in February and March; and are often checked or killed by the return of frost and cold after that period; and this has been evidently the case with the Platanus Occidentalis, American Plane, the one which has suffered so much of late, the other kinds remaining without any injury having occurred to them. The time these trees received their death-blow was in the spring of 1809, when it will be recollected, that we had a dreadful flood all over this kingdom; and that, during March and April, we had very mild weather, during which time these trees were greatly forwarded in germination (as were many other kinds from the same cause, and which suffered considerably at the time); this was succeeded by a very severe frost, which appears to have ruptured the sap vessels, so that the greater part of these trees have since died in consequence. In such an extraordinary season as this was, it would almost appear presumptuous in any person's attempting to explain the real cause of so mortifying a phenomenon; it is therefore only a matter of opinion. That the extreme moisture had been in a great measure the cause, I was firmly persuaded; but there is, moreover, proof that the cold had been its principal agent; for small trees of this kind have escaped, where they have been in thick plantations, protected by other kinds; whilst those growing nearly in the same spot, and not having the same protection, have been completely killed; and this has been the case with several in my garden. I am now speaking of small trees, under 10 feet high, of which I have lost many hundreds; but these of larger growth are, I believe, generally destroyed all over the country."

---

The first class of the National Institute has nominated M. VON HUMBOLDT to the place of Foreign Associate, vacant by the death of Mr. Cavendish.

---

Accouns from Santa Fé, in New Grenada, dated August 19, 1809, mention the death of the celebrated MUTIS, the friend of Linnæus, and one of the greatest botanists of the age. This venerable and worthy man had devoted upwards of fifty years to the examination of the vegetable productions of America. Attached at first as physician to the Viceroy, the count of Casa Flores, he began at his own expense to have drawings, made by native painters, formed by himself, for the *Flora of Bagota*. This grand work will be continued, and is greatly extended since he was appointed director of the botanical expedition of New Grenada. He had collected in his house considerable herbaries, more than 1500 coloured drawings of new plants, philosophical and astronomical instruments, and a collection of botanical works, inferior only to that of the illustrious President of the Royal Society of London.

Mr. Rea, one of Mutis's pupils, is the present director of the botanical garden of Madrid. His nephew, Don Sinforosa Mutis, has been commissioned by the government to complete the *Flora of Bagota*, for which no more than 566 descriptions of new species have been found drawn up by the deceased. Messrs. Mutis and Rixa, two distinguished artists, natives of Santa Fé, are finishing the numerous drawings that were were begun. M. Mutis, who in his old age had embraced the ecclesiastical profession, was equally distinguished for the variety and solidity of his attainments, and for the liberality and elevation of his sentiments. Previous to his death, he directed that his library, collections, and instruments, should be applied to the public use of his fellow-citizens.—Europe is indebted to him for the important discovery of the quinquina of New Grenada. The orange-coloured quinquina, of Santa Fe (*cinchona lancifolia*) which is not inferior in quality to the bark of Loxa, (*cinchona condaminea*) has become an important branch of commerce, at the ports of Carthagena, and Santa Martha.

---

In the ensuing spring, Dr. Thomas Jameson, of Cheltenham, will publish an Enquiry into the Physiological changes of the human body at its different ages, the diseases to which it is predisposed in each period of life, and the principles of longevity.

---

During last winter, a phenomenon, which would appear incredible, were it not attested by a great number of persons of known veracity, occurred in the vicinity of Placentia. On the 17th of January, red snow fell upon the mountains in this department, and especially upon that known by the name of Cento-croci. A coat of white snow had covered the tops of these mountains, when several peals of thunder, accompanied with lightning, were heard. From this moment, the snow that fell was red; this continued for some time, after which white snow again fell, so that the red was inclosed between two strata of white. In some places, this snow was only of the color of peach-blossom, but in others of deep red. Some of it was collected, and the water which it yielded, when melted, retained the same colour. The analysis of it by M. Guigotti, a chemist of Parma, promises interesting results. This phenomenon seems to furnish us with the means of explaining the showers of blood, which are mentioned by the ancients in their histories. We have already ascertained the existence of *pesinites*, or stones fallen from the atmosphere, which the Greeks and Latins have spoken of; and now it is impossible to deny the reality of showers of a blood-red color, which are described by the same authors.

---

From the New York Medical Repository.

*Sickness in the Army at New-Orleans, in* 1809.

GREAT mortality was suffered by the detachment of the American army encamped on the bank of the Mississippi, below New-Orleans. The troops were ordered to a place about fifteen miles to the south-east of the city. The ground selected by the quarter-master was low and swampy. A spot was cleared in the forest for their reception. Frequently, after a shower of rain, the camp was wet and muddy. In the rear were puddles of stagnant water. A luxuriant crop of vegetables grew in these, and putrified in the course of the season.

When

When the heats came on during the summer, the exhalations were rank and offensive.

Quartered on such damp ground, and breathing such a noxious atmosphere, the men soon became sickly. And this predisposition to disease was the more serious, inasmuch as the officers and soldiers came chiefly from more elevated and northerly climes, and had never been seasoned to southern service.

The situation of the army was the more calamitous, by reason of an omission to organize the medical staff, and to establish a regular hospital department. They who fell sick were not furnished with the usual accommodations. There were not even medicines in the camp to be dispensed to those who stood in need of them. Generally destitute of nurses, remedies, and attendance, the diseased recovered by strength of constitution or sunk under their complicated miseries.

But their condition was further aggravated. The beef and pork of their rations were remarked to be often in a corrupting and stinking state. Whether the salt had been too sparingly applied to them, or was of a weak and faulty quality, is not finally settled; but so it was, the meat was disgusting and unwholesome.

Fresh provisions were seldom afforded to the men; and the few that were issued were poor and scanty. There seemed to be no regular organization of the commissary's department.

As might have been predicted, the men became diseased, and died so rapidly, that orders were given to quit the station, and move the survivors to the higher country, near to Natchez. The prevailing forms of diseases in the camp were diarrhœas, dysenteries, remitting and intermitting fevers. In process of time, also, the scurvy mingled with the other maladies. This scorbutic diathesis manifested itself by swelled gums and loosened teeth. The soldiers, as surgeon Cutter declared to Dr. Mitchill, could frequently extract sound teeth from their gums with their fingers. Ulcers broke out in the mouth, tonsils and throat, of a most malignant character; and ate away all before them. The cheeks have been sometimes perforated quite through by this destructive ulceration; and even the large blood-vessels of the neck corroded so as to discharge their contents, and terminate life by sudden hæmorrhage.

In the House of Representatives an inquiry was instituted to investigate this miserable business; and Mr. Newton, from the committee appointed to inquire into the causes of the mortality which prevailed in the detachment of the army ordered for the defence of New-Orleans, made a long report, accompanied with various depositions and other papers. It concludes as follows:

"The committee, from a knowledge which they have acquired of the climate of New-Orleans and of the country surrounding it, and from the facts stated in the depositions, are of opinion that the mortality in the detachment ordered to New-Orleans is to be ascribed to the following causes:

"1. The detachment consisting of new levies.

"2. The insalubrity of the climate, the summer and autumn of 1809 being unusually sickly.

" 3. To the nature of the ground on which the detachment was encamped at Terre aux Bœuf, and the detention of it at that place during the whole of the summer, contrary, as the committee conceive, to the instructions contained in the letter of the Secretary of War, bearing date the 30th of April, 1809.

" 4. To the want of sound and wholesome provisions and of vegetables: the want of an hospital, and of hospital stores and medicines.

" 5. The excessive fatigues to which the troops were subjected in clearing, ditching, and draining the ground on which they were encamped.

" 6. To the want of repose during the night, owing to the troops not being provided with bars or nets, to protect them from the annoyance of musquitoes.

" 7. The want of cleanliness in the camp, the nature of the position rendering it almost impracticable to preserve it.

" 8. The sick and well being confined to the same tents, which neither protected them sufficiently from the heat of the sun, nor kept them dry from dews and rains."

---

*Curious Effect of Lightning upon a Lady's golden Chain and the Skin of her Neck.*

A REMARKABLE operation of the electric fluid is related in the " Journal des Dames et des Modes," for 20th June, 1809. On the 1st June, a house kept as a boarding-school for girls, at Bourdeaux, was struck by lightning; as was also one of the ladies. Being put to bed, she did not recover her voice for two hours after the accident, nor her recollection before the end of six hours. Nor had she the faintest remembrance of what had befallen her.

A chain of gold, which she wore around her neck, was melted and oxyded by the lightning. Her whole neck was covered with a black laced band. About her neck there are seven burns, resembling the marks produced by the application of a hot iron. Their surface was of a purple colour. A stripe passed in a zig-zag direction, from the left side of the bosom to the arm-pit of the same side, and there ended in a round spot equally purplish. On the left arm was found a similar stripe, extending below the bend of the arm where the part was covered by the clothing.

The purple hue resembled a skin coloured by a solution of gold, and could only be ascribed to the oxyd of that metal. The zig-zag stripes indicated the passage of the lightning, conducted by the gold, which was melted and oxyded in its passage. The round spot which terminates the stripe at the arm-pit, would seem to have been occasioned by a longer continuance of the gold, and of the fulminating agent upon that part.

### *Crawford's Translation of the "Fathers of Physic."*

John Crawford, M. D. of Baltimore, proposes a translation of Baron Haller's Latin edition of the "Artis Medicæ Principes." A paragraph from his printed prospectus will give an idea of this laudable and spirited design.

"The indefatigable and illustrious Haller has given a new edition "in Latin of Hippocrates, Aretæus, Alexander, Aurelianus, Celsus "and Rhazes, which is contained in ten volumes octavo. It is de- "signed to translate every thing contained in these which can con- "tribute to convey any useful instruction; but, in doing this, a slavish "regard to the language of the author will be dispensed with; im- "portant information will be alone regarded. Thus the quantity of "matter will be considerably reduced, and the expence proportionably "diminished. So modified, the work would be, perhaps, more ac- "ceptable than the original, even to those who possess, and are well "acquainted with its contents."

